# A potential role of salicylic acid in the evolutionary behavior of *Trichoderma* as a plant pathogen: from *Marchantia polymorpha* to *Arabidopsis thaliana*

**DOI:** 10.1007/s00425-022-04036-5

**Published:** 2022-11-28

**Authors:** Jorge Poveda, Patricia Abril-Urías, Julia Muñoz-Acero, Carlos Nicolás

**Affiliations:** 1 Department of Plant Production and Forest Resources, University Institute for Research in Sustainable Forest Management (iuFOR), University of Valladolid, Palencia, Spain; 2 Institute of Environmental Sciences, Plant Physiology Area, Universidad de Castilla-La Mancha, Toledo, Spain; 3 Department of Botany and Plant Physiology, Institute for Agrobiotechnology Research (CIALE), Universidad de Salamanca, Salamanca, Spain

**Keywords:** *Arabidopsis*, *Dryopteris affinis*, Evolution, *Marchantia polymorpha*, Plant defense pathways, Plant–fungi interaction

## Abstract

**Main conclusion:**

Recognition of the interaction of *Trichoderma* during the evolution of land plants plays a potential key role in the development of the salicylic acid defense pathway and the establishment of a mutualistic relationship.

**Abstract:**

*Marchantia polymorpha* is a common liverwort considered in recent years as a model plant for evolutionary studies on plant–microorganism interactions. Despite the lack of research, remarkable results have been reported regarding the understanding of metabolic and evolutionary processes of beneficial and/or harmful interactions, owing to a better understanding of the origin and evolution of different plant defense pathways. In this study, we have carried out work on the direct and indirect interactions (exudates and volatiles) of *M. polymorpha* with different species of the fungal genus *Trichoderma*. These interactions showed different outcomes, including resistance or even growth promotion and disease. We have analyzed the level of tissue colonization and defense-related gene expression. Furthermore, we have used the pteridophyte *Dryopteris affinis* and the angiosperm *Arabidopsis thaliana*, as subsequent steps in plant evolution, together with the plant pathogen *Rhizoctonia solani* as a control of plant pathogenicity. *Trichoderma virens*, *T. brevicompactum* and *T. hamatum* are pathogens of *M. polymorpha,* while exudates of *T. asperellum* are harmful to the plant. The analysis of the expression of several defense genes in *M. polymorpha* and *A. thaliana* showed that there is a correlation of the transcriptional activation of SA-related genes with resistance or susceptibility of *M. polymorpha* to *Trichoderma*. Moreover, exogenous SA provides resistance to the virulent *Trichoderma* species. This beneficial fungus may have had an evolutionary period of interaction with plants in which it behaved as a plant pathogen until plants developed a defense system to limit its colonization through a defense response mediated by SA.

## Introduction

*Marchantia polymorpha* represents the most widely distributed common liverwort in temperate regions. Taxonomically, it belongs to the Marchantiales subclass, including most of the 400 species, and in turn belongs to the Marchantiopsida class, the Marchantiophyta division and the Bryophyta sensu lato superdivision (Shimamura 2015). For almost 200 years, this species has been used as a model organism for the study of physiological and morphological changes in response to environmental factors. However, it is currently becoming increasingly important as a model plant (Shimamura 2015; Bowman 2016) due to its short life cycle (3–4 weeks from a spore to a mature thallus), ease of propagation and crossing, high frequency of transformation, haploidy and small genome size (approximately 280 Mb), low genetic redundancy and susceptibility to *Agrobacterium*-mediated transformation (Solly et al. 2017).

*Marchantia polymorpha* can also be used as a model plant to study the events that occurred during the transition from aquatic to terrestrial life, contributing to the understanding of the genetic bases of the evolutionary and developmental biology of land plants (Bowman et al. 2017). And, more recently, research has been conducted on this species as part of a new and interesting scientific area focused on the evolutionary interactions of molecular plant microbes (EvoMPMI) (Betsuyaku 2020). Although *M. polymorpha* has proven quite useful, few studies have been conducted on plant–fungi interactions involving *M. polymorpha*. Therefore, the use of this species as a model plant to study molecular plant pathology and plant–microorganism interactions, in the new scientific area of EvoMPMI, is gaining ground (Poveda 2020a).

*Trichoderma* is a genus of filamentous fungi that includes several widely studied species, some of which have been used as biological control agents due to their easy adaptation to diverse climatic and edaphic conditions and their different mechanisms of action. *Trichoderma* can protect the plant against different pathogens through mechanisms such as mycoparasitism (Guzmán-Guzmán et al. 2019), antibiosis (Estrada-Rivera et al. 2020) and competition with the pathogen for space, specific sites of infection and growth factors (Vinale et al. 2008). Likewise, the establishment of a *Trichoderma*-plant symbiotic relationship provides a series of indirect benefits such as the promotion of plant growth (Benítez et al. 2004; Contreras-Cornejo et al. 2018), increased tolerance against abiotic (Kashyap et al. 2017; Poveda 2020b) and biotic stresses caused by fungal pathogens (Poveda 2021a), nematodes (Poveda et al. 2020) and even insects (Poveda 2021b). For this symbiotic relationship to take place, *Trichoderma* has to establish a complex molecular dialogue with its host plant (Hermosa et al. 2012). *Trichoderma* acts as a symbiont capable of colonizing the roots, but without reaching the vascular bundle, limiting itself only to the outermost layers (Alonso-Ramírez et al. 2014). A comparative analysis of the genomes of several *Trichoderma* species has shown that mycoparasitism was the ancestral way of life for this genus. The subsequent colonization of the rhizosphere was probably due to the presence of pathogens in the soil and plant exudates (Kubicek et al. 2011, 2019).

The main goal of this work was to study the effect of different *Trichoderma* species on direct and indirect interaction with *M. polymorpha*; to determine the plant defense responses involved in this interaction, to check the similarities with more evolved vascular plants such as pteridophytes and angiosperms, and to increase our knowledge about the evolutionary development of plant defense responses against *Trichoderma*.

## Materials and methods

### Plants and fungi used

*Marchantia polymorpha* Tak-1 (subsp. *ruderalis*) was kindly provided by Dr. Isabel Monte from the laboratory of Dr. Roberto Solano (National Center for Biotechnology-CSIC, Madrid, Spain). The pteridophyte *Dryopteris affinis* sp. *affinis* was kindly provided by Dr. Elena María Fernández González (Area of Plant Physiology, University of Oviedo). Plants were continuously maintained in active asexual growth in Gamborg B5 (GMB) (Duchefa, Haarlem, The Netherlands) solid medium (agar 1%) in a growth chamber at 22 ºC, 40% relative humidity (RH) and a 16 h light/8 h dark photoperiod at 80–100 µmol photons m^−2^ s^−1^. *Arabidopsis thaliana* Columbia ecotype (Col-0) was obtained from the *Arabidopsis* collection Information Service (AIS), which is integrated within the European collection of the Nottingham *Arabidopsis* Stock Center (NASC).

Different species of the genus *Trichoderma* were used in this study, kindly provided by the Recognized Research Group (GIR) "Plant Pathology and Biological Control" of the Institute for Agrobiotechnology Research (CIALE), University of Salamanca. *T. parareesei* (named as T6), *T. atroviride* (named as T11), *T. asperellum* (named as T25), *T. harzianum* (named as T34), *T. koningii* (named as T77), *T. virens* (named as T87), *T. brevicompactum* (named as T120) and *T. hamatum* (named as T123) were the species used in this work. Furthermore, the fungus *R. solani*, anastomosis group 8 (AG8), was used as a broad host range pathogen, which was gifted by the Regional Diagnostic Center of the Regional Government of Castile and Leon (Salamanca, Spain). All fungi were grown on potato dextrose agar (PDA) medium (Sigma-Aldrich, St. Louis, MO, USA).

### Direct interaction in vitro

Fragments of approximately 1 cm^2^ of *M. polymorpha* thalli were placed inside Petri dishes containing GMB medium, with a total of 6 plants placed along the entire edge of each plate. After 3 weeks of growth in a culture chamber, under the conditions previously described, each Petri dish was inoculated with one of the fungal species used in the study by placing one agar cylinder loaded with mycelium from 2-week-old PDA dishes in the center of each plate. All plants and fungi were kept in a growth chamber for a total of 9 days.

For the *A. thaliana* assay, seeds were surface-sterilized by vigorous sequential shaking in 70% ethanol and 1% Tritón X-100 solutions for 20 min each, followed by washes in 2.5% sodium hypochlorite and 0.005% Tritón X-100 solutions for 10 min each. The seeds were then plated on Petri dishes containing GMB solid medium and kept in a growth chamber for 2 weeks.

Four seedlings of *A. thaliana* and *D. affinis* were transferred to Phytatray I boxes (Sigma) containing 40 mL of GMB solid medium. The boxes were kept in a growth chamber for 1 week in the case of *A. thaliana* and for 2 months in the case of *D. affinis*. These species were inoculated with the different fungal strains as previously indicated for *M. polmorpha*. The plants and fungi were again kept in a growth chamber for 7 days. All assays were maintained until *Trichoderma* completely colonized the entire surface of the culture medium, 9 days in the case of *M. polymorpha* and 7 days in the case of *A. thaliana* and *D. affinis*.

### Direct interaction on a growing substrate

For *M. polymorpha*, *D. affinis* and *A. thaliana*, Phytatray II boxes (Sigma) containing 100 mL of peat and vermiculite (1:3), 15 mL of sterile water and 3 mL of liquid GMB were used, following the AtCube system method (Poveda 2022a). Fragments of approximately 1 cm^2^ of *M. polymorpha* thalli and seedlings from *A. thaliana* and *D. affinis* were placed inside Phytatray II boxes. Six plants per box were analyzed and placed in a growth chamber for 3 weeks (*M. polymorpha*), 8 weeks (*D. affinis*) or 1 week (*A. thaliana*), at 22 °C, 40% relative humidity (RH) and a 16 h light/8 h dark photoperiod at 80–100 μmol photons m^−2^ s^−1^.

Fungal inoculation was carried out in the same way for all experimental assays, following the method described by Poveda (2021c). *Trichoderma* spores were harvested from 2-week-old PDA dishes and each plant was inoculated with 1 mL of a conidial suspension containing 2 × 10^7^ spore mL^−1^. For *R. solani*, all mycelium present on a 2-week-old PDA dish was collected, crushed and diluted to an absorbance of 0.17 mL^−1^ at 520 nm and applied to each plant. The plants and fungi were again kept in a growth chamber for 30 days (*M. polymorpha*) or 10 days (*D. affinis* and *A. thaliana*).

### Indirect interaction through fungal exudates

To evaluate the impact of fungal exudates on *M. polymorpha*, two types of 30-mL liquid cultures were prepared: GMB and GMB + 0.3% (w/v) of *M. polymorpha* tissue (previously lyophilized and ground in a Falcon tube). To each tube, either a 1 mL of a spore solution, diluted to a 2 × 10^7^ mL^−1^ in the case of *Trichoderma* or a homogeneous suspension of *R. solani* mycelium with 0.17 absorbance at 520 nm was added. The 0.3% (w/v) *M. polymorpha*-medium was prepared to check for the production of fungal metabolites only in the presence of plant tissues. All tubes were maintained at 28 °C and 200 rpm for 48 h. The tubes were then centrifuged and the medium was harvested by filtration through a sterile 0.22 µm filter. Fragments of approximately 1 cm^2^ of *M. polymorpha* thalli were placed in Petri dishes containing GMB and 1 mL of the fungal filtrate. Seven plants per dish were used and placed in a growth chamber for 2 weeks. This method was initially developed using tomato callus and fungal filtrates from the pathogenic fungus *Leptosphaeria maculans* and has proven to be effective for carrying out in vitro studies (Poveda 2022b).

### Indirect interaction by volatiles

For the analysis of the effect of *Trichoderma* spp. and *R. solani* volatile compounds on *M. polymorpha*, four open 55 cm Ø Petri dishes were placed into a 150 cm Ø Petri dish. Two of the plates, containing PDA medium, were inoculated with either *Trichoderma* spp. or *R. solani* by placing a circle of agar from previous cultures. In the remaining two plates, containing GMB medium, three fragments of *M. polymorpha* were seeded. This 150 cm Ø Petri dish was placed in a growth chamber for 2 weeks.

### Plant growth analysis

The quantification of the plant biomass of *M. polymorpha* was carried out using the different pictures taken at the end of each assay, following the method described by Poveda (2020c). The plants were photographed inside Phytatray II boxes or in the Petri dishes and the images obtained were analyzed by quantifying the percentage of existing plants using the MulticolorEngine software (TinEye, Toronto, Ontario, Canada) (https://labs.tineye.com/color/). In this way, we quantified each plant according to the space it occupied within a 2D plane. The same method was used for *D. affinis* and *A. thaliana*.

### Vitality test

A vitality test was carried out as described by Poveda (2020c). One plant from a *M. polymorpha*-*Trichoderma* direct interaction assay was collected per Petri dish (8 plants per condition and repetition). Due to the activity of the mitochondrial respiratory chain of living plant cells, the reduction of triphenyltetrazolium chloride (TTC) to the red-colored insoluble triphenylformazan (TF) occurs (Ruf and Brunner 2003). Therefore, only living cells can reduce TTC to TF. In total, 100 mg of fresh tissue were transferred to 1 mL of 1% TTC in triplicate and incubated for 48 h at 37 °C. After incubation, 200 mg of Ballotini Glass Balls (100 mg with a diameter of 0.15–0.25 mm and 100 mg with a diameter of 1 mm) were added to each sample in 1.5 mL Eppendorf tubes, which were shook vigorously using a 20-s pulse in a Silamat S6 (Ivoclar Vivadent, Madrid, Spain). After centrifuging the samples for 15 min at 16,770*g*, each supernatant was removed and 1 mL of isopropanol was added per tube. Subsequently, the samples were shaken again in a Silamat and centrifuged. The absorbance of each supernatant at 620 nm was quantified, being an indirect measurement of the vitality of *M. polymorpha* tissue.

### Analysis of *Trichoderma* colonization

For the molecular analysis of colonization, one plant was collected per Petri dish (8 per condition and repetition) from the *M. polymorpha*–*Trichoderma *in vitro direct interaction. Each plant was superficially washed with sterile water and immediately frozen in liquid nitrogen and ground with a mortar and pestle. The quantification of *Trichoderma* DNA in *M. polymorpha* tissue was performed by qPCR as previously described by Poveda et al. (2019a), with some modifications. DNA was extracted from the tissues using a cetyl-trimethyl-ammonium bromide (CTAB) extraction method, as reported previously (Dellaporta et al. 1983). A mix was prepared in a 10 μL volume using 5 μL of Brilliant SYBR Green QPCR Master Mix (Roche, Penzberg, Germany), 10 ng of DNA, the forward and reverse primers at a final concentration of 100 nM and nuclease-free PCR-grade water to adjust the final volume. The *actin* gene of *Trichoderma* and the *Elongation Factor 1* (*EF1*) gene of *M. polymorpha* were used; their corresponding primer pairs are indicated in Table 1. Amplifications were performed in an ABI PRISM 7000 Sequence Detection System (Applied Biosystems, Foster City, CA, USA) programmed for 40 cycles under the following conditions: denaturation, 95 °C for 15 s; annealing, 60 °C for 1 min; extension, 72 °C for 1 min. Each PCR was performed in triplicate by using the DNA extracted from 3 pools of tissue collected from 8 plants per condition. Cycle threshold (Ct) values served to calculate the amount of fungal DNA using standard curves. The values obtained for *Trichoderma* DNA were referred to the amount of *M. polymorpha* DNA.

**Table 1:** Primers used in this work

Code	Sequence (5′–3′)	Use	References
T-Act-F	ATGGTATGGGTCAGAAGGA	*Actin* to *Trichoderma* quantification	Poveda (2021c)
T-Act-R	ATGTCAACACGAGCAATGG		
Mp-EF1-F	AAGCCGTCGAAAAGAAGGAG	Endogenous *M. polmorpha* gene	Yoshikawa et al. (2018)
Mp-EF1-R	TTCAGGATCGTCCGTTATCC		
Mp-ICS-F	GACTATGAGGAGGTTTCTTTCC	Synthesis gene of SA in *M. polmorpha*	Gimenez-Ibanez et al. (2019)
Mp-ICS-R	GCTACATTTACTGCAAGTAGGG		
Mp-PR1-F	TAACAACTGTCAGCTGAAGACC	Response gene of SA in *M. polmorpha*	Gimenez-Ibanez et al. (2019)
Mp-PR1-R	CTTCCAGACAACCTGAGTGTAA		
Mp-LOX1-F	GGCATATGGATTTACACACAGCGAG	Synthesis gene of JA in *M. polmorpha*	Kanamoto et al. (2012)
Mp-LOX1-R	CCGGATCCTAGATGGAAATGCTCCAAG		
Mp-COI1-F	AGGACAGAAGGCACTGAAGTTC	Response gene of JA in *M. polmorpha*	Monte et al. (2018)
Mp-COI1-R	CTGCTTCTCAGAAACAGTCATGC		
At-ACT-F	CTCCCGCTATGTATGTCGCC	Endogenous *A. thaliana* gene	Poveda et al. (2019b)
At-ACT-R	TTGGCACAGTGTGAGACACAC		
At-ICS1-F	GATCTAGCTAACGAGAACGG	Synthesis gene of SA in *A. thaliana*	Poveda et al. (2019b)
At-ICS1-R	CATTAAACTCAACCTGAGGGAC		
At-PR1-F	GGCTAACTACAACTACGCTG	Response gene to SA in *A. thaliana*	Poveda et al. (2019b)
At-PR1-R	GGCTTCTCGTTCACATAATTC		
At-LOX1-F	GTAAGCTCTGATGTTACTGATTC	Synthesis gene of JA in *A. thaliana*	Poveda et al. (2019b)
At-LOX1-R	CTGCGGTTAACGACGTGATTG		
At-VSP2-F	GTTAGGGACCGGAGCATCAA	Response gene to JA in *A. thaliana*	Poveda et al. (2019b)
At-VSP2-R	TCAATCCCGAGCTCTATGATGTT		

### Gene expression studies

The analysis of the expression of defense-related genes in *M. polymorpha* tissue and *A. thaliana* roots was carried out by RT-qPCR, using the methodology described by Poveda (2020c, 2021c), with some modifications. For *M. polymorpha*, one plant was collected per Phytatray II box (8 per condition and repetition) from the *M. polymorpha*-*Trichoderma* direct interaction on a growing substrate, 10- (when the growth of the fungus is still visible) and 30-days (when the disease symptoms observed were very severe) post fungal inoculation. Each plant was superficially washed with sterile water and immediately frozen in liquid nitrogen and ground using a mortar and pestle. For *A. thaliana*, one plant was collected per Phytatray II box (8 per condition and repetition) from the *A. thaliana*-*Trichoderma* direct interaction on growing substrate 10 days post fungal inoculation. At this timepoint, the plant started the formation of flower stalks, and the highest root fungal colonization take place, as we have checked based on our experience in the *Trichoderma*–*A. thaliana* interaction. All root material was washed with water to remove all remaining substrate, immediately frozen with liquid nitrogen and ground using a mortar and pestle.

RNA extraction was carried out with the TRI reagent (Ambion, Austin, TX, USA), following the manufacturer’s instructions. cDNA was synthesized from 2 µg of RNA, which was treated with DNase RQ1 (Promega Biotech Ibérica, Alcobendas, Spain), and then used for reverse transcription with an oligo (dT) primer with the Transcriptor First Strand cDNA Synthesis Kit (Takara Bio, Tokyo, Japan), following the manufacturer’s protocol. Gene expression was analyzed by RT-qPCR, using an ABI PRISM 7000 Sequence Detection System with Brilliant SYBR Green QPCR Master Mix (Stratagene, La Jolla, CA, USA). All PCR reactions were performed in triplicate using the cDNA obtained from three pools of tissue from 8 plants per condition, in a total volume of 10 µL for 40 cycles under the following conditions: denaturation, 95 °C, 30 s; annealing, 60 °C, 1 min; extension, 72 °C, 1 min. Threshold cycles (Cts) were determined using the 7000 SDS System Software (Applied Biosystems) and CT values were calculated using the *M. polymorpha EF1* gene as an endogenous control. The primers used are given in Table 1: genes of the isochorismate synthase (*ICS*), pathogenesis-related protein 1 (*PR-1*), synthesis and response genes to salicylic acid (SA), respectively, and lipoxygenase 1 (*LOX1*), and coranatine-insensitive 1 (*COI1*) and vegetative storage protein (*VSP2*), synthesis and response genes to jasmonic acid (JA), respectively.

### Exogenous application of salicylic acid

To determine the effect of exogenous application of SA on *M. polymorpha*-*Trichoderma* interaction, the in vitro direct interaction was carried out again by irrigating each plant-thalli superficially with 20 µL of 0.25 mM SA (Sigma-Aldrich) immediately before fungal inoculation.

### Statistical analysis

The statistical analysis of the data was carried out using the Statistix 8.0 software. One-way ANOVA using Tukey’s multiple range test at *P* < 0.05 was used for pairwise comparisons; the different letters indicate the significant differences.

## Results

### *Trichoderma*-plant direct interaction in vitro

**Fig. 1:**
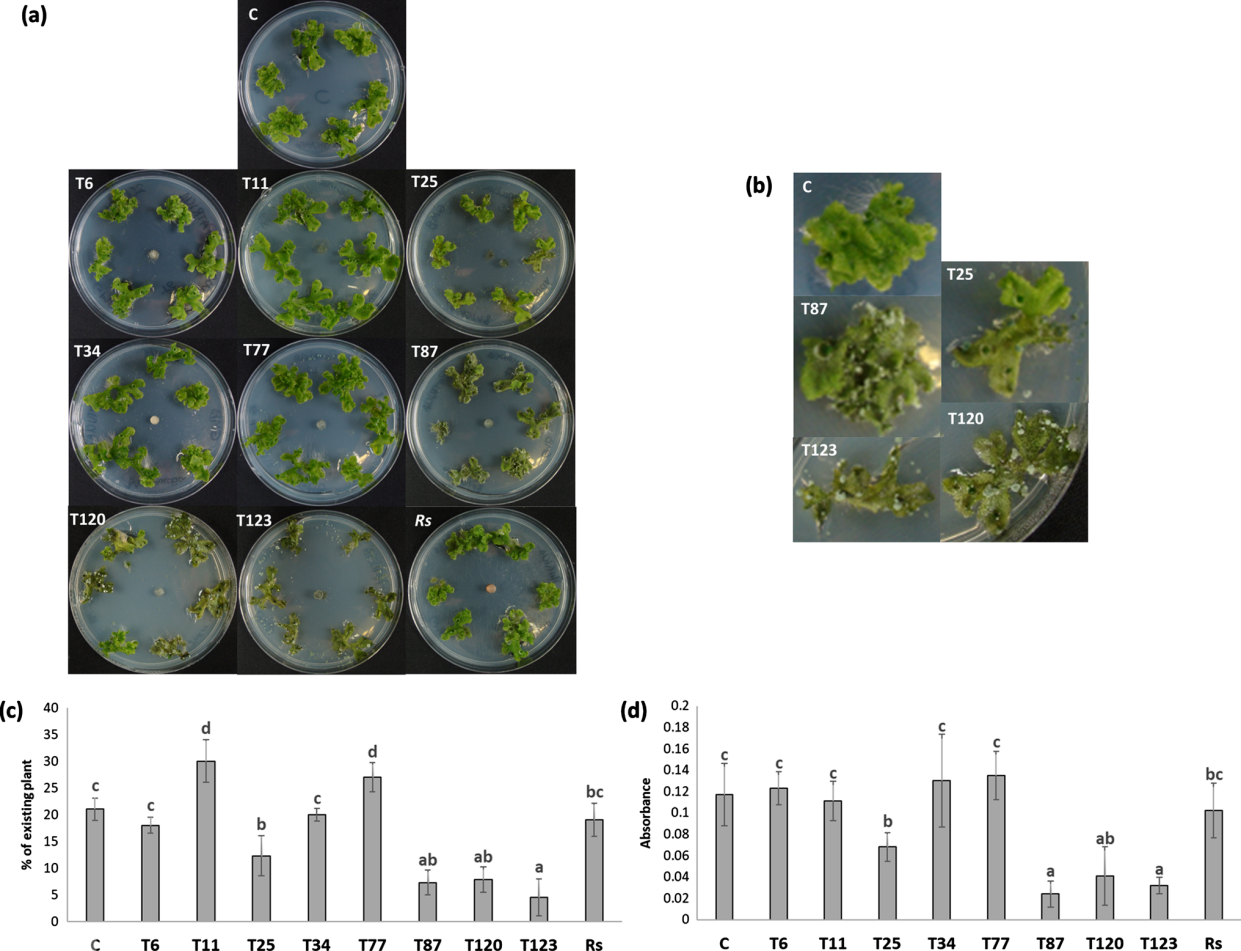
*Trichoderma*–*M. polymorpha* interaction on in vitro culture. Photographs of Petri dishes (**a**) and individual details of plants (**b**) were taken 9 at dpi, and plant biomass analyzed by visual quantity of the plant (**c**) of *M. polymorpha* without fungi (C) and inoculated with *T. parareesei* (T6), *T. atroviride* (T11), *T. asperellum* (T25), *T. harzianum* (T34), *T. koningii* (T77), *T. virens* (T87), *T. brevicompactum* (T120), *T. hamatum* (T123) and *R. solani* (*Rs*). Data are the mean of three biological replicates for each condition with the corresponding standard deviation, and for each biological replicate and condition, eight dishes were used. In tissue vitality by the triphenyltetrazolium chloride (TTC) test (**d**) in tissues of *M. polymorpha*, the absorbance at 620 nm (TTC test) was analyzed. Data are the mean of three biological replicates for each condition with the corresponding standard deviation, and for each biological replicate and condition, tissues from eight plants were used. One-way analysis of variance (ANOVA) was performed, followed by the Tukey’s test. Different letters represent significant differences (*P* < 0.05)

*Trichoderma* species T25, T87, T120 and T123 produced symptoms of plant damage 9 days post-inoculation (dpi) in the in vitro* M. polymorpha*-*Trichoderma* interaction assay (Fig. 1), a result that was not observed with the rest of *Trichoderma* species or with the plant pathogen *R. solani* (Fig. 1a, b, d and e). By contrast, the application of T11 and T77 seemed to have a positive effect on *M. polymorpha* growth (Fig. 1a). Through the quantification of plant growth in a 2D plane, the application of T25, T87, T120 and T123 lead to a significant reduction in plant biomass, compared to the non-inoculated plants, which was even more significant after the application of T123. Furthermore, inoculations using T11 and T77 induced a significant increase in *M. polymorpha* biomass (Fig. 1c).

Regarding the vitality of *M. polymorpha* tissue (Fig. 1d), the application of T25 and T120 significantly reduced the vitality of *M. polymorpha* tissue compared to the non-inoculated plants; this reduction was even more significant after the application of T87 and T123 (Fig. 1d).

**Fig. 2:**
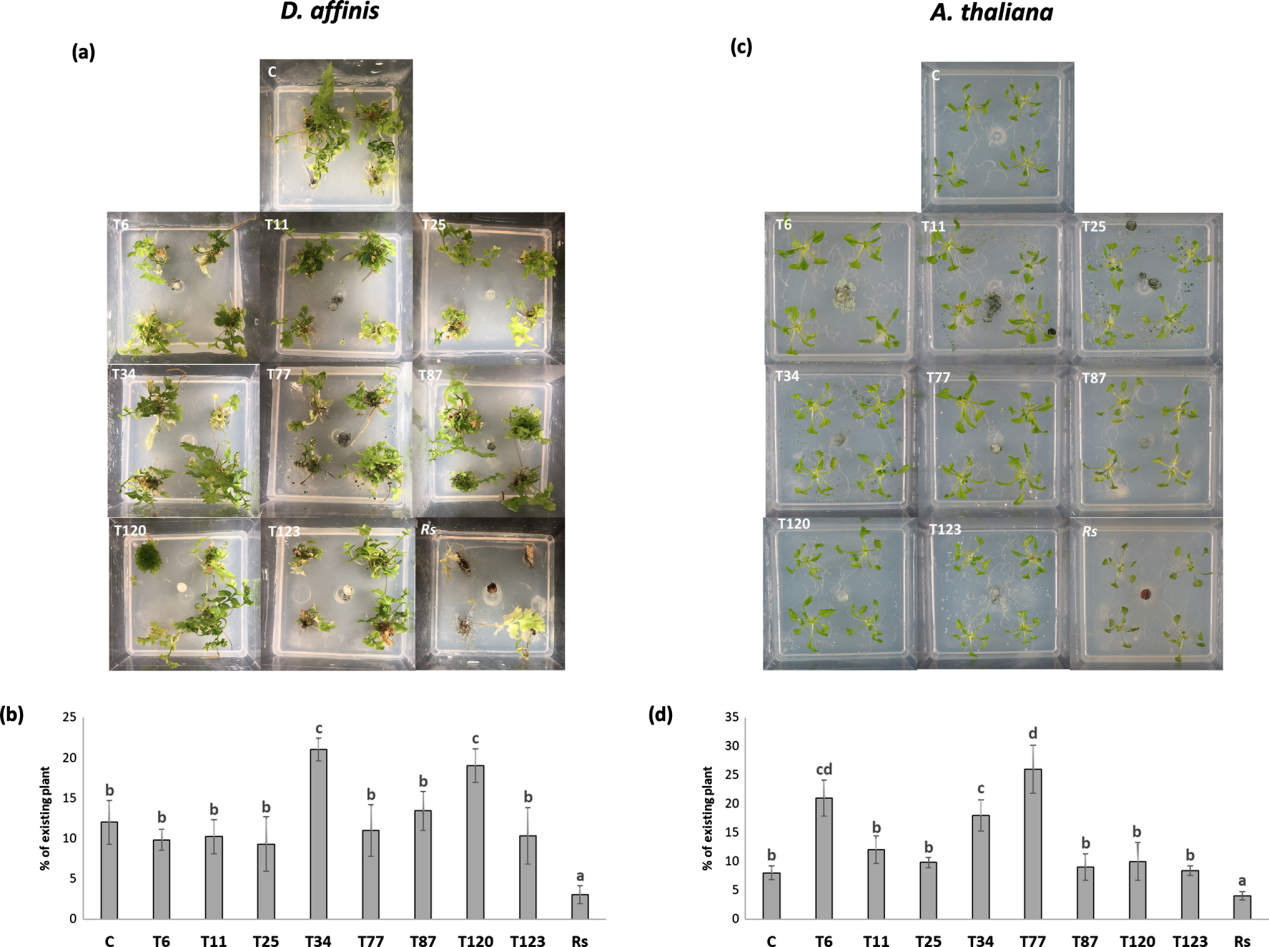
*Trichoderma*–*D. affinis* (**a**, **b**) and *Trichoderma*–*A. thaliana* (**c**, **d**) interaction on in vitro culture. Photographs of *D. affinis* (**a**) and *A. thaliana* (**c**) in Phytatray II boxes were taken at 7 dpi, and plant biomass analyzed by visual quantity of *D. affinis* (**b**) and *A. thaliana* (**d**) of plants without fungi (C) and inoculated with *T. parareesei* (T6), *T. atroviride* (T11), *T. asperellum* (T25), *T. harzianum* (T34), *T. koningii* (T77), *T. virens* (T87), *T. brevicompactum* (T120), *T. hamatum* (T123) and *R. solani* (*Rs*). Data are the mean of three biological replicates for each condition with the corresponding standard deviation, and for each biological replicate and condition, five boxes were used. One-way analysis of variance (ANOVA) was performed, followed by the Tukey’s test. Different letters represent significant differences (*P* < 0.05)

Conversely, in the in vitro* D. affinis*–*Trichoderma* interaction (Fig. 2a, b) assay, the application of different *Trichoderma* species did not cause symptoms of damage to the pteridophyte, which were observed after inoculation with *R. solani* (Fig. 2a). These symptoms were accompanied by a significant decrease in plant biomass after *R. solani* inoculation compared to the non-inoculated plants, while a significant increase in plant biomass was observed after the application of T34 and T120.

The inoculation of *R. solani* resulted in disease symptoms in *A. thaliana* plants, while no disease symptoms were observed after inoculation with different *Trichoderma* species (Fig. 2c). These results were consistent with the significant reduction in the biomass of *A. thaliana* plants provoked by *R. solani* and its significant increase after T6, T34 and T77 inoculation (Fig. 2d).

The quantification of *Trichoderma* colonization in *M. polymorpha* tissue showed that the best colonizers were T87, T120 and T123 (Table 2).

**Table 2:** *M. polymorpha*-*Trichoderma* colonization on in vitro culture

*Trichoderma* specie	Plant			Fungi			Ratio^3^
	Ct	SD	Qty^1^	Ct	SD	Qty^2^	
T6	19.38	0.08	2.35	23.07	0.12	0.42	0.18 ± 0.04^a^
T11	18.72	0.13	2.98	22.61	0.25	1.01	0.34 ± 0.01^b^
T25	19.63	0.02	2.11	23.07	0.18	0.42	0.20 ± 0.03^a^
T34	19.17	0.19	2.55	23.08	0.14	0.41	0.16 ± 0.06^a^
T77	19.37	0.12	2.36	22.72	0.22	0.87	0.37 ± 0.05^b^
T87	19.32	0.04	2.41	21.84	0.19	2.00	0.83 ± 0.07^c^
T120	18.72	0.16	2.98	21.45	0.05	2.50	0.84 ± 0.14^c^
T123	19.08	0.09	2.63	21.18	0.24	2.45	0.93 ± 0.05^c^

*Trichoderma* species *T. parareesei* (T6), *T. atroviride* (T11), *T. asperellum* (T25), *T. harzianum* (T34), *T. koningii* (T77), *T. virens* (T87), *T. brevicompactum* (T120), *T. hamatum* (T123) were used

Quantification of *Trichoderma* DNA in *M. polymorpha* was performed by qPCR

^1^Quantity of *M. polymorpha* DNA (ng) referred to *EF1* gene

^2^Quantity of *Trichoderma* DNA (ng) referred to *Actin* gene

^3^Proportion of fungal DNA vs. plant DNA. Values are the means of three plant-pools (eight plants each one) from three independent experiments with the corresponding standard deviations. One-way analysis of variance (ANOVA) was performed, followed by the Tukey’s test. Different letters represent significant differences (*P* < 0.05)

### *Trichoderma*-plant direct interaction on a growing substrate

**Fig. 3:**
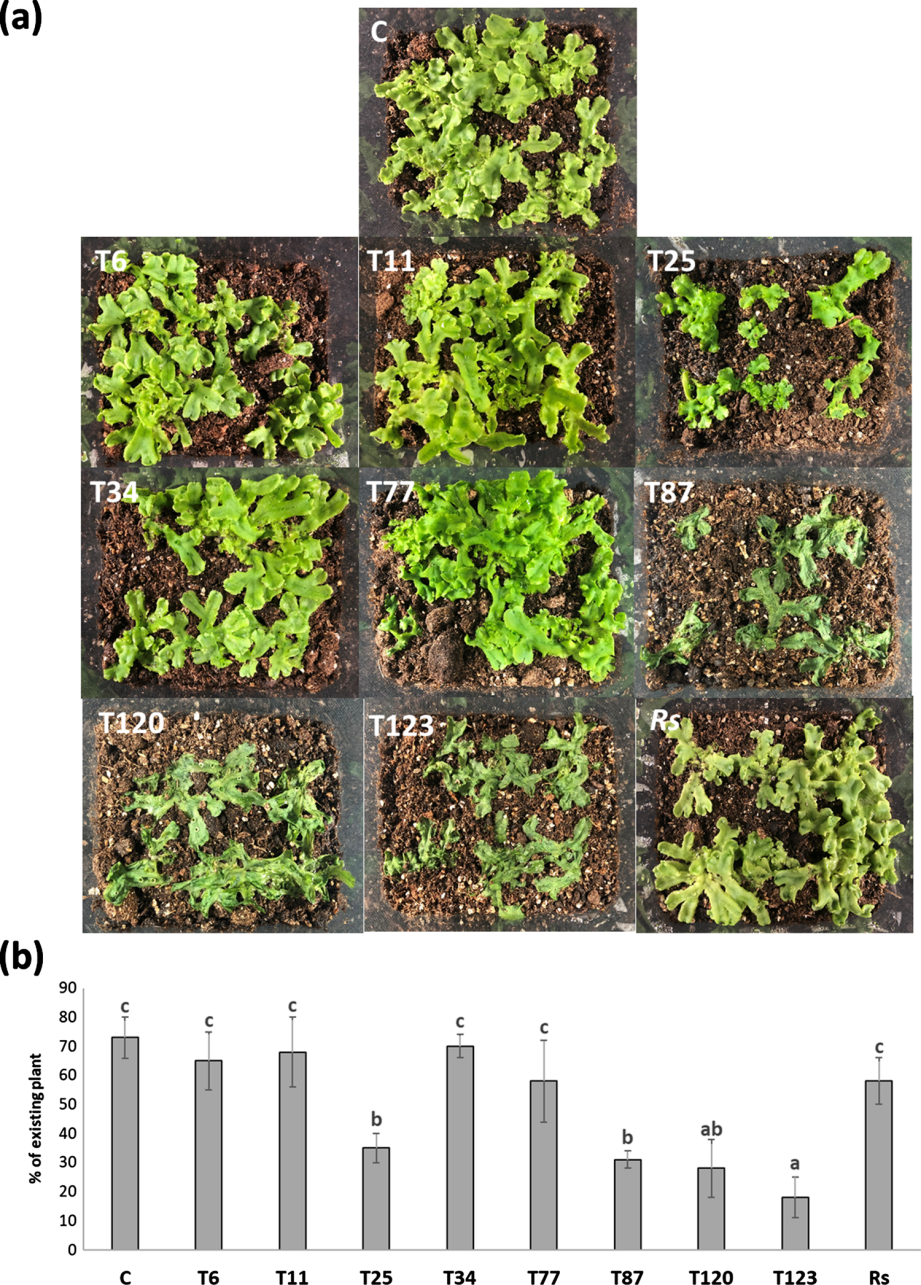
*Trichoderma*-*M. polymorpha* interaction on growing substrate. Photographs of Phytatray II boxes (**a**) were taken at 30 dpi, and plant biomass analyzed by visual quantity of the plant (**b**) of *M. polymorpha* without fungi (C) and inoculated with *T. parareesei* (T6), *T. atroviride* (T11), *T. asperellum* (T25), *T. harzianum* (T34), *T. koningii* (T77), *T. virens* (T87), *T. brevicompactum* (T120), *T. hamatum* (T123) and *R. solani* (*Rs*). Data are the mean of three biological replicates for each condition with the corresponding standard deviation, and for each biological replicate and condition, eight six boxes were used. One-way analysis of variance (ANOVA) was performed, followed by the Tukey’s test. Different letters represent significant differences (*P* < 0.05)

No harmful effects were observed in *M. polymorpha* after *R. solani* inoculation or after inoculation with T6, T11, T25, T34 and T77 (Fig. 3a). On the contrary, T87, T120 and T123 inoculation produced disease symptoms in *M. polymorpha*. These results were accompanied by a significant decrease in *M. polymorpha* growth compared to non-inoculated plants or those inoculated with the other fungal strains (Fig. 3b).

**Fig. 4:**
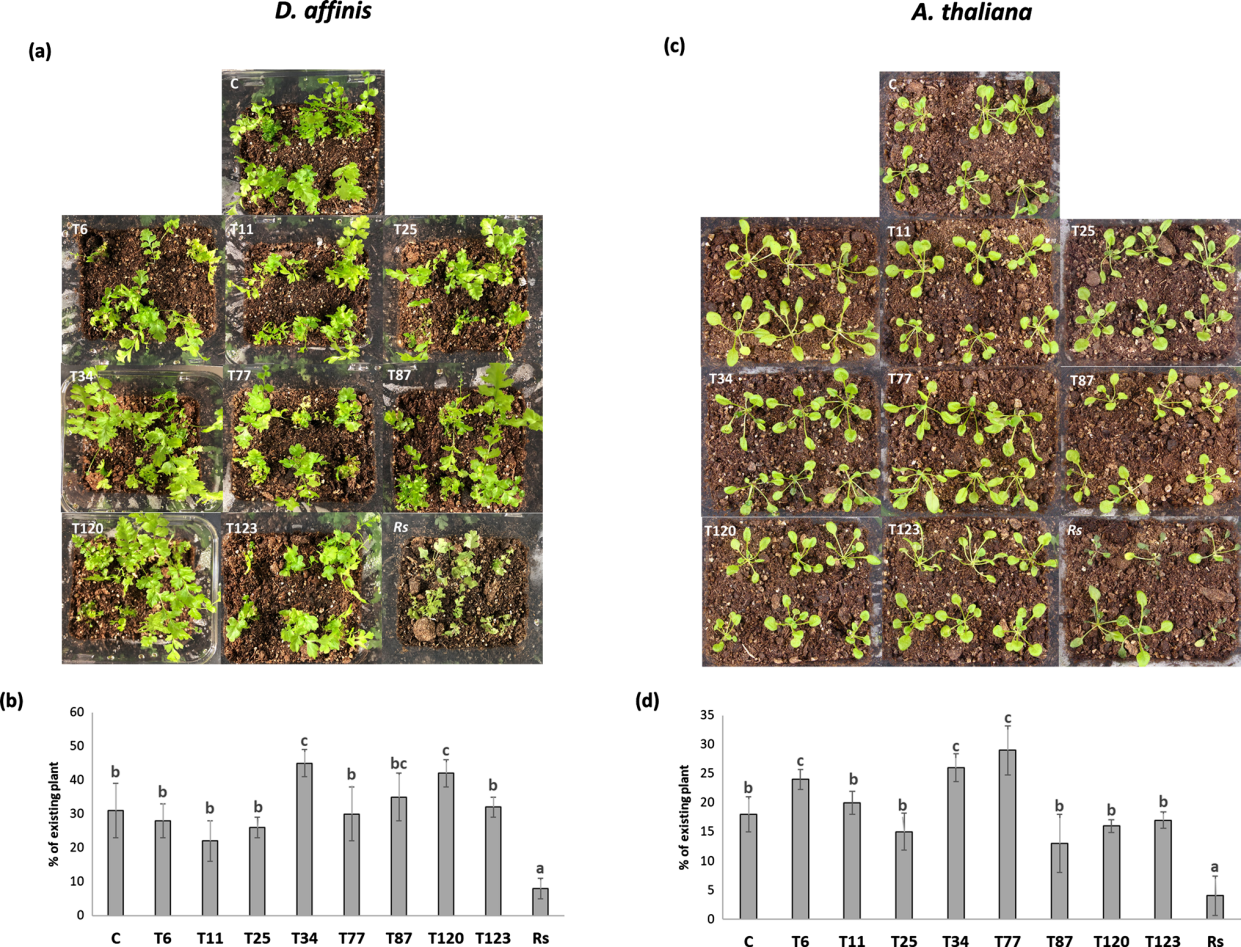
*Trichoderma*–*D. affinis* (**a**, **b**) and *Trichoderma*–*A. thaliana* (**c**, **d**) interaction on growing substrate. Photographs of *D. affinis* (**a**) and *A. thaliana* (**c**) in Phytatray II boxes were taken at 10 dpi, and plant biomass analyzed by visual quantity of *D. affinis* (**b**) and *A. thaliana* (**d**) of plants without fungi (C) and inoculated with *T. parareesei* (T6), *T. atroviride* (T11), *T. asperellum* (T25), *T. harzianum* (T34), *T. koningii* (T77), *T. virens* (T87), *T. brevicompactum* (T120), *T. hamatum* (T123) and *R. solani* (*Rs*). Data are the mean of three biological replicates for each condition with the corresponding standard deviation, and for each biological replicate and condition, six boxes were used. One-way analysis of variance (ANOVA) was performed, followed by the Tukey’s test. Different letters represent significant differences (*P* < 0.05)

The inoculation of *Trichoderma* species did not result in disease symptoms in the case of the pteridophyte *D. affinis*. Moreover, the application of T34 and T120 had a beneficial effect on growth promotion, whereas *R. solani* inoculation was clearly detrimental to *D. affinis* (Fig. 4a, b).

In the case of *A. thaliana*, *Trichoderma* inoculation did not seem to have a negative effect, whereas disease symptoms were observed after *R. solani* challenge (Fig. 4c). The quantification of plant biomass confirmed the negative effect of *R. solani* on *Arabidopsis* plants, as well as the beneficial effects on plant growth of different *Trichoderma* species, such as T6, T34 and T77 (Fig. 4d).

### Defense-gene expression

**Fig. 5:**
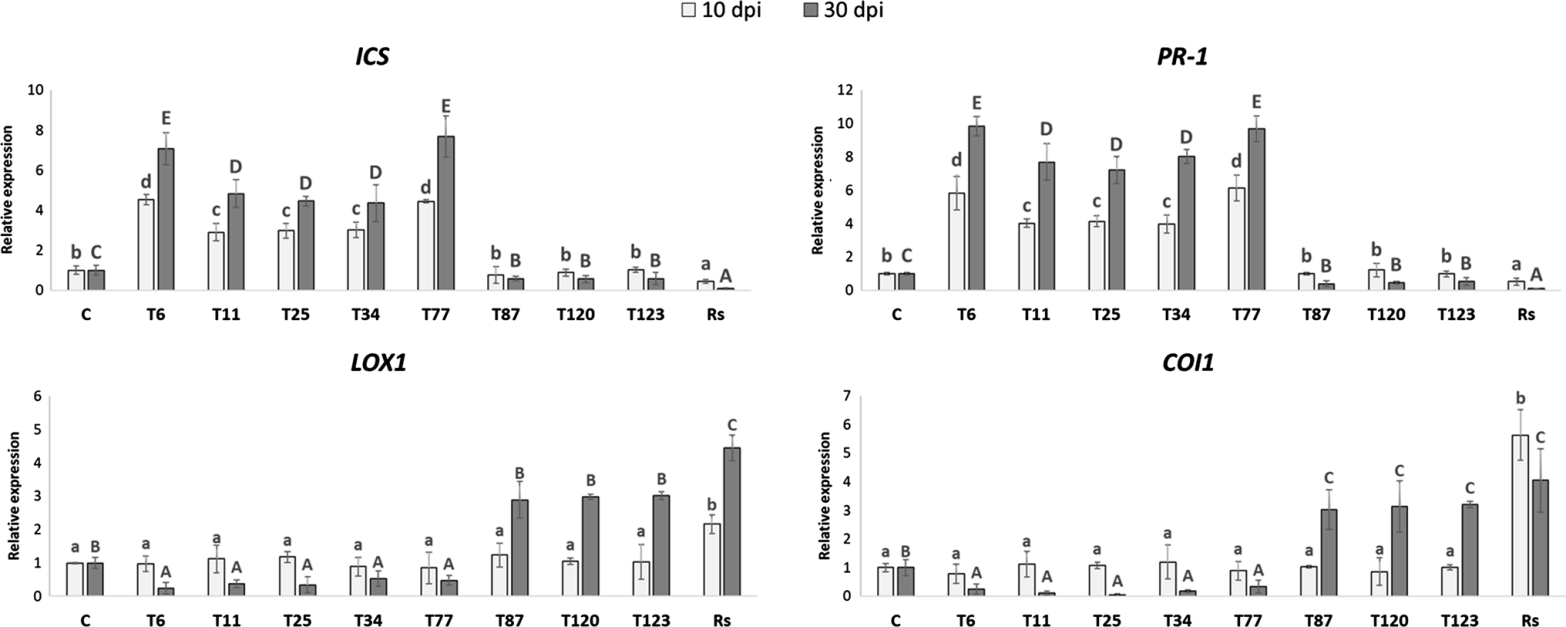
Quantitative reverse transcription polymerase chain reaction (RT-qPCR) analysis of the expression of some defense genes in *M. polymorpha*-tissues without fungi-inoculation (C) and inoculated with *T. parareesei* (T6), *T. atroviride* (T11), *T. asperellum* (T25), *T. harzianum* (T34), *T. koningii* (T77), *T. virens* (T87), *T. brevicompactum* (T120), *T. hamatum* (T123) and *R. solani* (*Rs*). The expression of the genes isochorismate synthase (*ICS*), pathogenesis-related protein 1 (*PR-1*), lipoxygenase 1 (*LOX1*) and coranatine-insensitive 1 (*COI1*) was quantified at 10 and 30 dpi. Values correspond to relative measurements against plants without fungi-inoculation (2^–ΔΔCt^ = 1). The *M. polimorpha EF1* gene was used as an internal reference gene. Data are the mean of three biological replicates for each condition with the corresponding standard deviation, and for each biological replicate and condition, tissues from eight plants were used. One-way analysis of variance (ANOVA) was performed, followed by the Tukey’s test. Different letters represent significant differences (*P* < 0.05) between the different conditions, lowercase letters on data from 10 dpi and capital letters on data from 30 dpi

The expression of SA-related genes, such as *ICS* related to SA-biosynthesis and *PR-1*, related to SA-action, increased significantly in *M. polymorpha* plants inoculated with T6, T11, T25, T34 and T77, both at 10 and 30 dpi. By contrast, no changes were observed in the expression of JA-related genes (*LOX1*, involved in JA-biosynthesis and *COI1*, involved in JA-action) compared with the non-inoculated plants. In addition, a significant decrease in SA-related genes and a significant increase in JA-related genes were detected after inoculation with T87, T120 and T123, as well as in the case of the pathogenic fungi *R. solani* (Fig. 5).

**Fig. 6:**
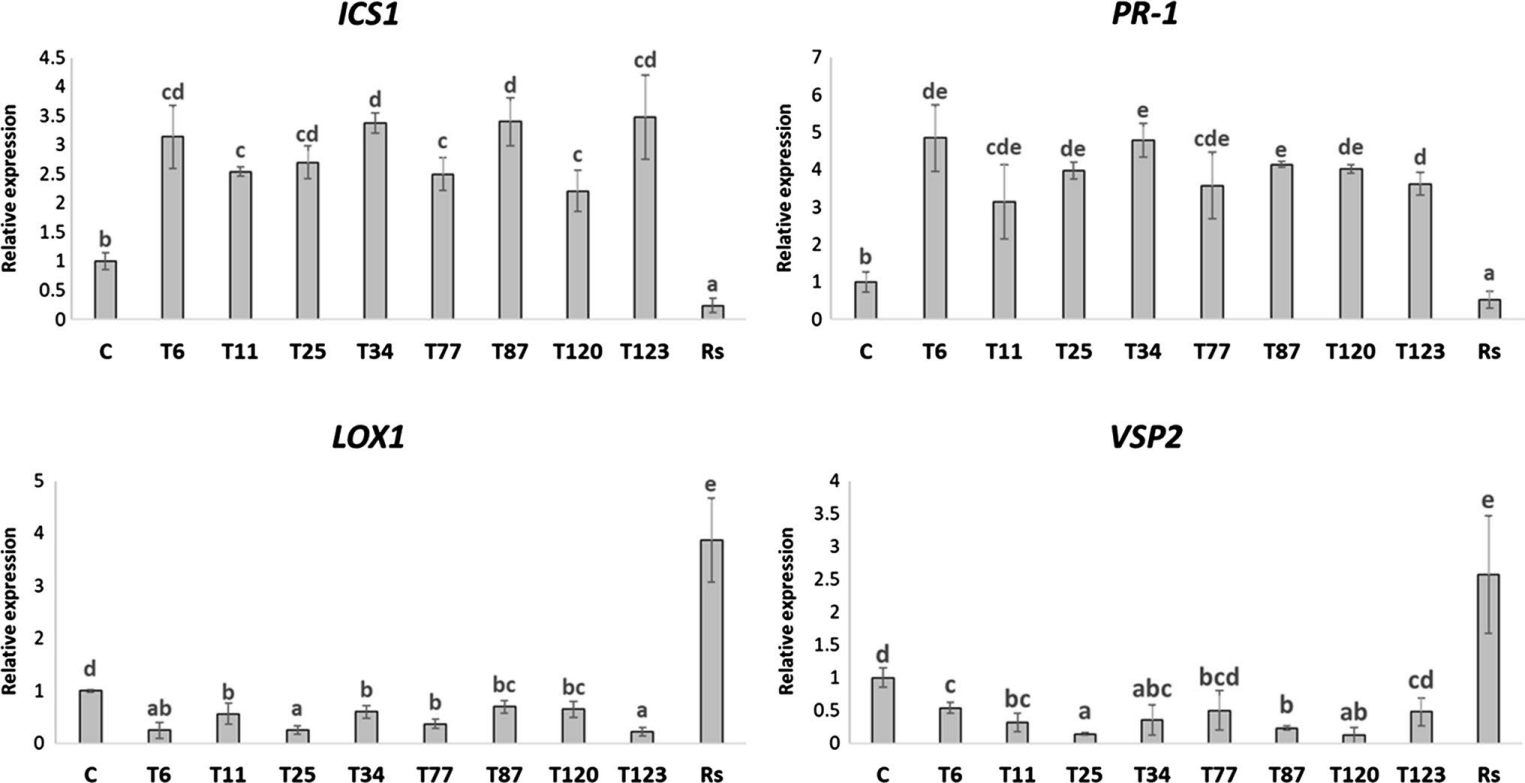
Quantitative reverse transcription polymerase chain reaction (RT-qPCR) analysis of the expression of some defense genes in *A. thaliana*-roots without fungi-inoculation (C) and inoculated with *T. parareesei* (T6), *T. atroviride* (T11), *T. asperellum* (T25), *T. harzianum* (T34), *T. koningii* (T77), *T. virens* (T87), *T. brevicompactum* (T120), *T. hamatum* (T123) and *R. solani* (*Rs*). The expression of the genes isochorismate synthase 1 (*ICS1*), pathogenesis-related protein 1 (*PR-1*), lipoxygenase 1 (*LOX1*) and vegetative storage protein (*VSP2*) was quantified. Values correspond to relative measurements against plants without fungi-inoculation (2^–ΔΔCt^ = 1). The *A. thaliana Actin* gene was used as an internal reference gene. Data are the mean of three biological replicates for each condition with the corresponding standard deviation, and for each biological replicate and condition, roots-pools from eight plants were used. One-way analysis of variance (ANOVA) was performed, followed by the Tukey’s test. Different letters represent significant differences (*P* < 0.05) between the different conditions

In the case of *A. thaliana*, *Trichoderma*-root inoculation produced a significant increase in SA-related genes and a significant decrease in the expression levels of JA-related genes. Moreover, root infection with R. *solani* provoked a significant decrease in the expression of SA-related genes and a significant increase in the expression of JA-related genes (Fig. 6).

### *Trichoderma*–*M. polymorpha* indirect interaction

**Fig. 7:**
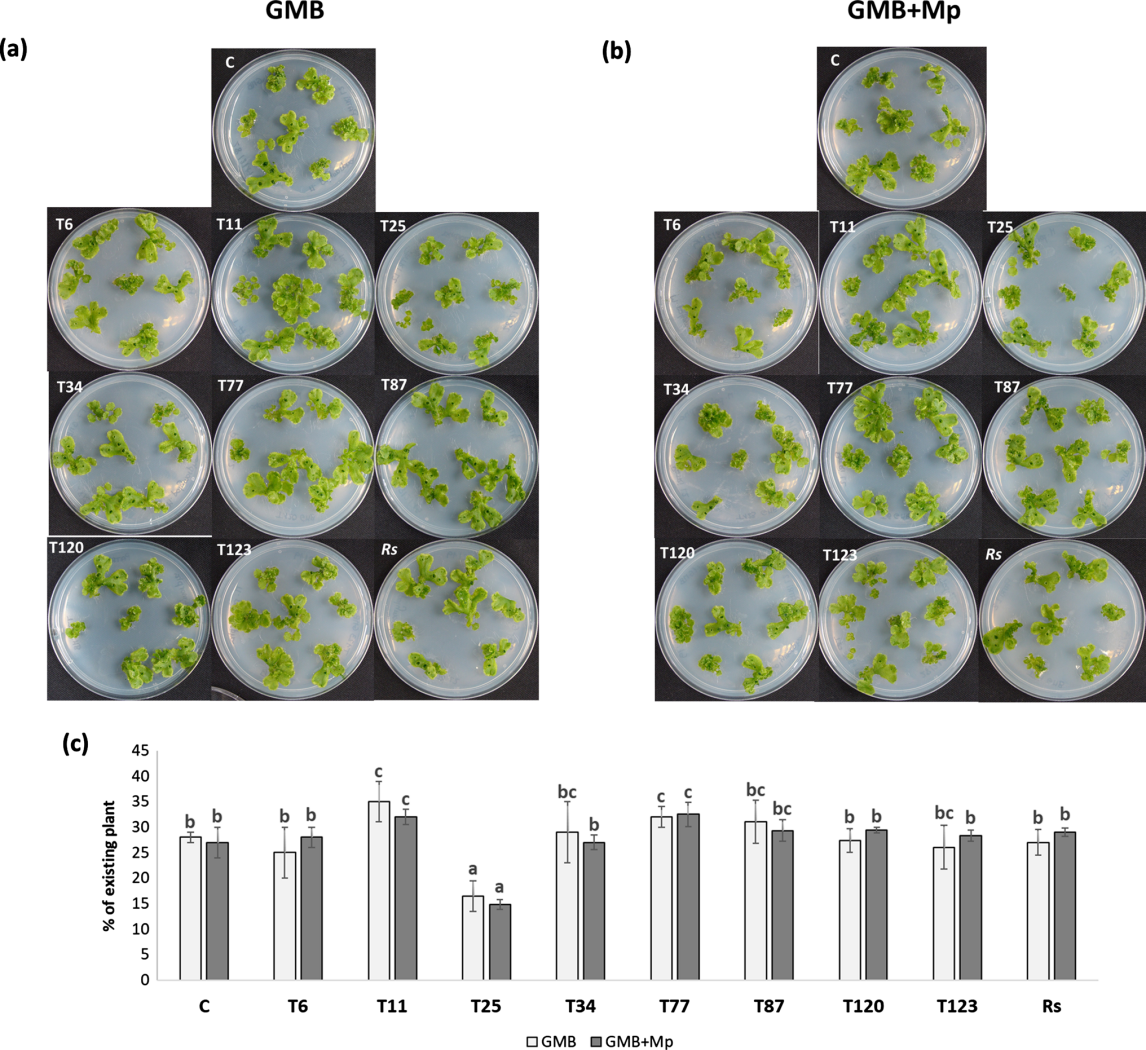
*Trichoderma*–*M. polymorpha* indirect interaction by exudates. Photographs of Petri dishes (**a** and **b**) were taken 2 weeks after contacting exudates-plants, and plant biomass analyzed by visual quantity of the plant (**c**) of *M. polymorpha* without fungi (C) and inoculated with *T. parareesei* (T6), *T. atroviride* (T11), *T. asperellum* (T25), *T. harzianum* (T34), *T. koningii* (T77), *T. virens* (T87), *T. brevicompactum* (T120), *T. hamatum* (T123) and *R. solani* (*Rs*). Plants contacted with exudates from fungal liquid culture in GMB (**a**) and in GMB-0.3% W/V *M. polymorpha*-tissues (**b**). Data are the mean of three biological replicates for each condition with the corresponding standard deviation, and for each biological replicate and condition, eight dishes were used. One-way analysis of variance (ANOVA) was performed, followed by the Tukey’s test. Different letters represent significant differences (*P* < 0.05)

The application of fungal exudates did not lead to the appearance of disease symptoms in any of the treatments (Fig. 7a, b). However, the analysis of plant biomass showed that the application of exudates from T25 significantly reduced the growth of *M. polymorpha* compared with plants without exudate application. In the case of the application of exudates from T11 and T77, a significant increase in plant growth was observed (Fig. 7c).

**Fig. 8:**
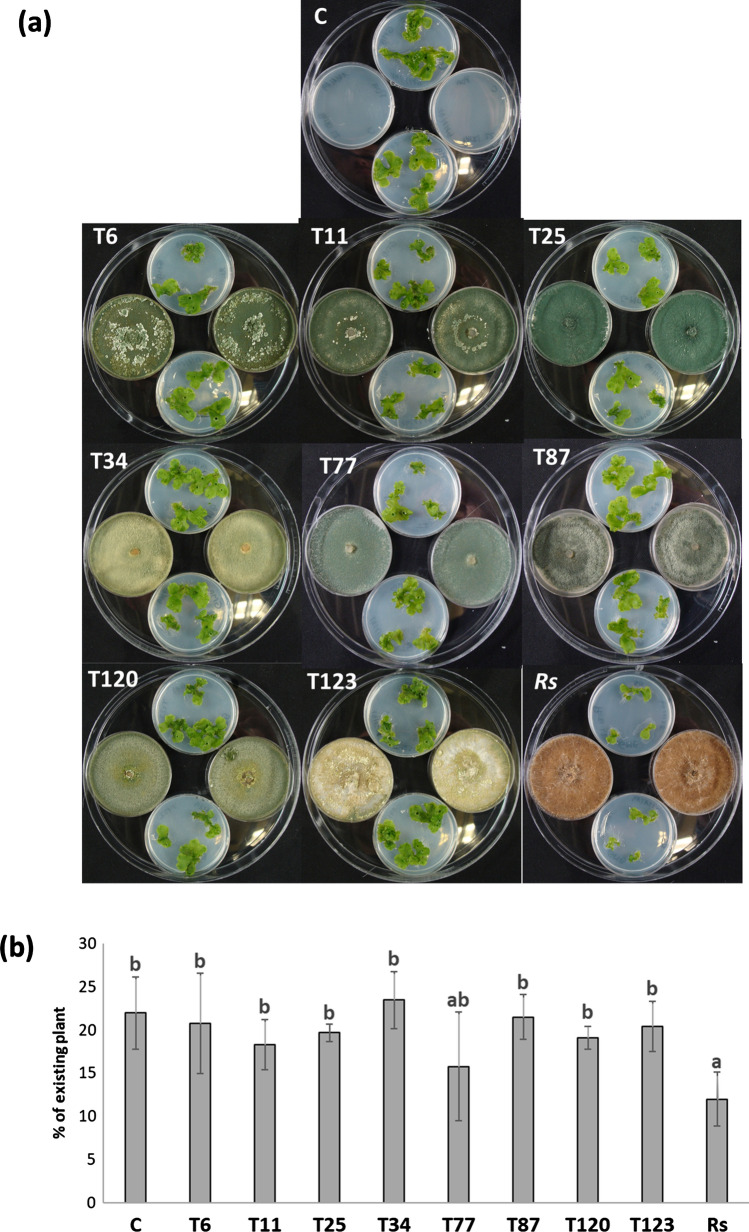
*Trichoderma*–*M. polymorpha* indirect interaction by volatiles. Photographs of Petri dishes (**a**) were taken 2 weeks in fungal-volatiles-contacting, and plant biomass analyzed by visual quantity of the plant (**b**) of *M. polymorpha* without fungi (C) and inoculated with *T. parareesei* (T6), *T. atroviride* (T11), *T. asperellum* (T25), *T. harzianum* (T34), *T. koningii* (T77), *T. virens* (T87), *T. brevicompactum* (T120), *T. hamatum* (T123) and *R. solani* (*Rs*). Data are the mean of three biological replicates for each condition with the corresponding standard deviation, and for each biological replicate and condition, ten dishes were used. One-way analysis of variance (ANOVA) was performed, followed by the Tukey’s test. Different letters represent significant differences (*P* < 0.05)

The application of fungal volatiles from different *Trichoderma* strains and *R. solani* did not produce disease symptoms in *M. polymorpha*. However, a significant decrease in plant growth was observed in *M. polymorpha* in contact with *R. solani* volatiles, compared to the untreated plants (Fig. 8b).

### SA exogenous application

**Fig. 9:**
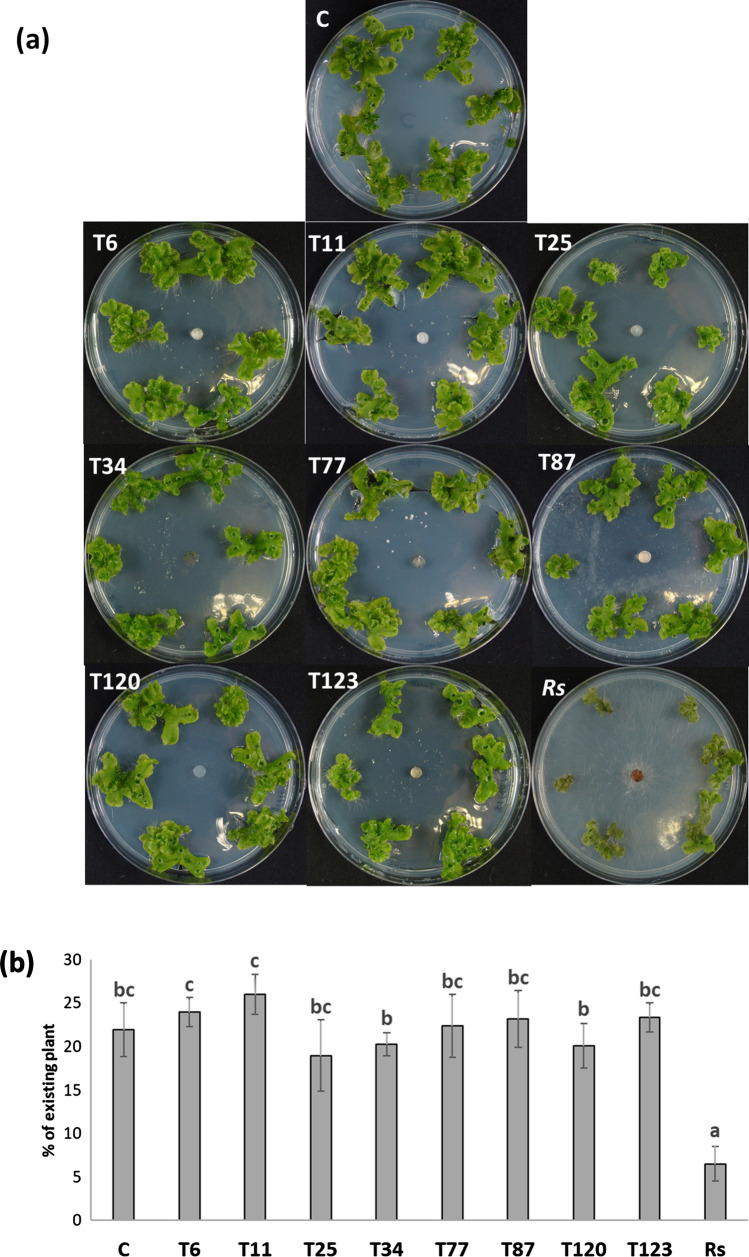
*Trichoderma*–*M. polymorpha* interaction on in vitro culture with exogenous application of SA 0.25 mM. Photographs of Petri dishes (**a**) were taken at 9 dpi, and plant biomass analyzed by visual quantity of the plant (**b**) of *M. polymorpha* without fungi (C) and inoculated with *T. parareesei* (T6), *T. atroviride* (T11), *T. asperellum* (T25), *T. harzianum* (T34), *T. koningii* (T77), *T. virens* (T87), *T. brevicompactum* (T120), *T. hamatum* (T123) and *R. solani* (*Rs*). Data are the mean of three biological replicates for each condition with the corresponding standard deviation, and for each biological replicate and condition, eight dishes were used. One-way analysis of variance (ANOVA) was performed, followed by the Tukey’s test. Different letters represent significant differences (*P* < 0.05)

After SA exogenous application in *M. polymorpha* plants, non-inoculated or inoculated with the different fungi, disease symptoms were only observed in plants inoculated with *R. solani* (Fig. 9a). This result was consistent with plant biomass quantification. The inoculation with *R. solani* was the only fungal treatment that resulted in a reduction in plant growth, compared with the non-inoculated plants (Fig. 9b).

## Discussion

Currently, there are only a few examples of microorganisms pathogenic to *M. polymorpha*, which makes this species rather interesting from the perspective of its use as a model plant in evolutionary interactions of molecular plant microbes (EvoMPMI) (Poveda 2020a). Several studies have been performed regarding the interaction of beneficial fungi, such as arbuscular mycorrhizal fungi, but not for *M. polymorpha* (Ligrone et al. 2007). Moreover, even possible adverse effects have not been reported (Poveda 2020c). The analysis of the endophytic fungal diversity from different samples of *M. polymorpha* revealed the presence of aggressive plant pathogens, such as *Colletotrichum truncatum*, which appear to have no harmful or beneficial effects to *M. polymorpha* (Nelson et al. 2018).

*Trichoderma* is a well-known genus of filamentous fungi beneficial to vascular plants, which has been widely used in agriculture (Ghazanfar et al. 2018; Mendoza-Mendoza et al. 2018). However, in this work, several species, such as *T. virens*, *T. brevicompactum* and *T. hamatum*, were tested for their potential to act as liverwort pathogens in direct interaction, since these species have been described as being parasitic on plant pathogenic fungi, as plant growth promoters, as activators of systemic plant resistance and capable of enhancing tolerance to abiotic stresses (Studholme et al. 2013; Trushina et al. 2013; Babu et al. 2014; Jogaiah et al. 2018; Mahesh et al. 2018; Racić et al. 2018). Other species such as *T. atroviride* and *T. koningii* have been found to have beneficial effects on *M. polymorpha* growth, being similar to those described for vascular plants including tomato (Macías-Rodríguez et al. 2018), rapeseed (Maag et al. 2014), radiate pine (Regliński et al. 2012) and rice (Lauinio et al. 2020).

As expected, most of the *Trichoderma* species tested have a positive effect on *A. thaliana*, as previously described for other plant species such as tomato (Rubio et al. 2014), rapeseed (Poveda et al. 2019b), maize (Vargas et al. 2009), as well as *A. thaliana* (Poveda et al. 2019b). The absence of a positive effect caused by the other *Trichoderma* species applied may result from the fact that the biostimulant effect depends on the species assayed, the amount of inoculum used and the plant species (Fiorentino et al. 2018). Similarly, the inoculation with *T. harzianum* and *T. brevicompactum* increased plant growth in *D. affinis*,

After the analysis of fungal colonization in the direct interaction of *M. polymorpha* and *Trichoderma*, we examined whether the *Trichoderma* species producing the disease symptoms (*T. virens*, *T. brevicompactum* and *T. hamatum*) were those with the highest level of fungal colonization (Fig. 1 and Table 2). Furthermore, the analysis of the expression of defense-related genes showed that no changes in SA-related genes were detected after 10 days of interaction in these three species, while an increase in the expression levels of JA-related genes was detected (Fig. 5). The *Trichoderma* species that did not exhibit a pathogenic effect against *M. polymorpha* (*T. parareesei*, *T. atroviride*, *T. harzianum*, *T. koningii* and *T. virens*) induced an SA-response, while a decrease in JA-related genes was observed after 30 days of interaction (Fig. 5). These results highlight the key role of SA in plant-*Trichoderma* interaction. In *Arabidopsis* plants with impaired SA biosynthesis, *Trichoderma* was able to colonize the vascular bundles of the plant and behave as a systemic pathogen, starting with root rot (Alonso-Ramírez et al. 2014). In this study, we have shown that the different *Trichoderma* species applied have no harmful effect on the *A. thaliana* plants colonized, as expected. In all cases, *Trichoderma* induced the expression of SA-related genes whereas no changes in JA-related genes were observed (Fig. 6). Thus, the activation of the SA pathway in plant tissues seems to play a key role in the molecular dialogue established between *Arabidopsis* and *Trichoderma* for avoiding uncontrolled colonization of its tissues. Exogenous application of SA to *M. polymorpha* (Fig. 9) prevents disease symptoms produced by *T. virens, T. brevicompactum* and *T. hamatum*, underlining again the main role of SA signaling in the plant defense response during *Trichoderma*-plant interaction. SA pathway plays an important role against biotrophic pathogens, and it has been proposed that this mechanism of action may be an ancient and evolutionarily pathway conserved between bryophytes and angiosperms and, therefore, likely appeared in the common ancestor of land plants (Giménez-Ibáñez et al. 2019). SA-biosynthetic and signaling genes are present in *Marchantia* (Bowman et al. 2017), other liverworts (Drabkova et al. 2015) and other bryophytes (Ponce de León et al. 2012), but not in algae (Giménez-Ibáñez et al. 2019). Given our results, *M. polymorpha*, a basal land plant, seems unable to recognize the chemical signals derived from the interaction with some *Trichoderma* species. The ability of different *Trichoderma* species to produce different type of metabolites could explain why *Marchantia* is not able to recognize microbe-specific molecules known as microbe-associated molecular patterns (MAMPS) derived from the pathogenic *Trichoderma* species and could explain the behavior of *Marchantia* when challenged with different *Trichoderma* species. This mechanism would prevent the activation of the SA pathway and lead to the subsequent invasion by these *Trichoderma* species that behave as plant pathogens; however, more studies are needed to understand exactly how this molecular dialogue takes place. At a higher level of the evolutionary scale, including pteridophytes (*D. affinis*) and angiosperms (*A. thaliana*), this mechanism of defense must have been completely developed, since none of the *Trichoderma* species used in this work produced disease symptoms (Fig. 4). This statement would have allowed the establishment of a mutualistic interaction between *Trichoderma* and plants.

Interestingly, *T. asperellum* caused disease symptoms in the direct interaction in vitro but not on a growth substrate (Fig. 1), while the colonization level was one of the lowest compared with other *Trichoderma* strains. However, the plant defense response was similar to other non-pathogenic strains. This could suggest that the detrimental effect of T25 is not due to direct interaction. Exposure of *M. polymorpha* plants to *Trichoderma* exudates would explain the mechanism by which *T. asperellum* would act negatively on plant biomass (Fig. 7).

As expected, *R. solani* acts as a pathogen for *A. thaliana*, as previously reported (Foley et al. 2013; Li et al. 2019), as well as for *D. affinis* (Fig. 4). However, no harmful effect for this pathogen was observed after *M. polymorpha* inoculation (Fig. 3). Moreover, the analysis of the expression of SA- and JA-responsive genes showed a similar pattern to that observed in the pathogenic *Trichoderma* strains. A significant reduction of *ICS1* and *PR-1* (SA-related), as well as a significant increase in *LOX1* and *COI1* (JA-related) levels, was observed after 10 and 30 days after fungal inoculation (Fig. 5). This SA-JA balance has an important role in the synthesis and accumulation of different defense chemical compounds (Gahtori and Chaturvedi 2011; Mewari and Kumar 2011). This result proved that the correct defense response against the pathogenic *Trichoderma* strains is through the SA and JA pathways against *R. solani*. The latter result is reinforced by the fact that exogenous application of SA, which would inhibit JA response, produced disease symptoms in *M. polymorpha* after *R. solani* inoculation. Similar results were observed in *Marchantia* after *Irpex lacteus* infection (Matsui et al. 2020). This SA/JA antagonism represents a pivotal role in plant defense responses, which may have arisen in the evolution of bryophytes such as *M. polymorpha* (Thaler et al. 2012; Berens et al. 2017). Despite this, we found that the volatiles produced by *R. solani* had only a detrimental effect on *M. polymorpha* plants, while in *A. thaliana* the volatiles enhanced growth and accelerated development (Cordovez et al. 2017). Microbial volatile organic compounds (MVOCs) can have general or very specific biological functions in some plant species. In this sense, there are pathogens whose MVOCs can have extremely beneficial effects on plants, promoting their growth, activating their systemic defenses, stimulating antifungal, oomycetidal and nematicidal activities, and even repel insect pests (Poveda 2021d).

In sum, our study has revealed how several *Trichoderma* species can behave as pathogens of *M. polymorpha*, but not of *D. affinis* or *A. thaliana*. This *Trichoderma* plant–pathogen behavior is a consequence of non-recognition by the host and the subsequent absence of response mediated by SA in *M. polymorpha*. Evolutionarily, it has been determined how *Trichoderma* has shifted from an ancestral way of life based on mycoparasitism to being a symbiont-plant thanks to the presence of exudates and pathogenic fungi in the rhizosphere (Kubicek et al. 2011, 2019). We hypothesize that between both evolutionary stages, *Trichoderma* behaved as a plant pathogen until SA-mediated plant-defense response was developed by land plants. Once the plant became able to recognize *Trichoderma*, a molecular dialogue may have been generated with the subsequent establishment of a mutualistic relationship between both organisms.

### Author contribution statement

JP, PAU and JMA conducted the laboratory work; JP and CN conceived and designed the experiments; JP, PAU, JMA and CN analyzed the data; JP and CN wrote the paper.

## Data Availability

All data generated or analyzed during this study are included in this published article.

## Abbreviations

COI1: Coronatine-insensitive 1
dpi: Days post-inoculation
GMB: Gamborg B5 medium
ICS: Isochorismate synthase
JA: Jasmonic acid
LOX1: Lipoxygenase 1
SA: Salicylic acid
